# The African-centric P47S Variant of *TP53* Confers Immune Dysregulation and Impaired Response to Immune Checkpoint Inhibition

**DOI:** 10.1158/2767-9764.CRC-23-0149

**Published:** 2023-07-11

**Authors:** David C. Stieg, Joshua L. D. Parris, Tyler Hong Loong Yang, Gauri Mirji, Sarah Kim Reiser, Nivitha Murali, Madison Werts, Thibaut Barnoud, David Y. Lu, Rahul Shinde, Maureen E. Murphy, Daniel T. Claiborne

**Affiliations:** 1 Program in Molecular and Cellular Oncogenesis, The Wistar Institute, Philadelphia, Pennsylvania.; 2 Program in Immunology, Microenvironment, and Metastasis, The Wistar Institute, Philadelphia, Pennsylvania.; 3 Department of Biochemistry and Molecular Biology, Medical University of South Carolina, Charleston, South Carolina.; 4 Vaccine and Immunotherapy Center, The Wistar Institute, Philadelphia, Pennsylvania.

## Abstract

The tumor suppressor *TP53* is the most frequently mutated gene in cancer and is mutationally inactivated in 50% of sporadic tumors. Inactivating mutations in *TP53* also occur in Li Fraumeni syndrome (LFS). In addition to germline mutations in *TP53* in LFS that completely inactivate this protein, there are many more germline mutant forms of *TP53* in human populations that partially inactivate this protein: we call these partially inactivating mutations “hypomorphs.” One of these hypomorphs is a SNP that exists in 6%–10% of Africans and 1%–2% of African Americans, which changes proline at amino acid 47 to serine (Pro47Ser; P47S). We previously showed that the P47S variant of p53 is intrinsically impaired for tumor suppressor function, and that this SNP is associated with increased cancer risk in mice and humans. Here we show that this SNP also influences the tumor microenvironment, and the immune microenvironment profile in P47S mice is more protumorigenic. At basal levels, P47S mice show impaired memory T-cell formation and function, along with increased anti-inflammatory (so-called “M2”) macrophages. We show that in tumor-bearing P47S mice, there is an increase in immunosuppressive myeloid-derived suppressor cells and decreased numbers of activated dendritic cells, macrophages, and B cells, along with evidence for increased T-cell exhaustion in the tumor microenvironment. Finally, we show that P47S mice demonstrate an incomplete response to anti-PD-L1 therapy. Our combined data suggest that the African-centric P47S variant leads to both intrinsic and extrinsic defects in tumor suppression.

**Significance:**

Findings presented here show that the P47S variant of *TP53* influences the immune microenvironment, and the immune response to cancer. This is the first time that a naturally occurring genetic variant of *TP53* has been shown to negatively impact the immune microenvironment and the response to immunotherapy.

## Introduction

The tumor suppressor p53 plays a critical role in the cellular response to nearly all forms of stress, including oncogenic, genotoxic, and metabolic stress (1–3). In response to such stress, p53 becomes posttranslationally stabilized and activated, and subsequently regulates several tumor suppressive pathways that include apoptosis, ferroptosis, cell-cycle arrest, and senescence (4–6). It is generally accepted that p53’s function as a transcription factor underlies these responses (7), although p53 also has a transcription-independent role in cell death at the mitochondria (8–10). Not surprisingly, the *TP53* gene is subject to mutational inactivation in the majority of tumors, rendering many of these cancers with poor prognosis and poor response to conventional cancer therapies (11).

In addition to mutational inactivation, the *TP53* gene also possesses a large number of germline missense variants that impact the function of this protein (12–14). A nonsynonymous SNP at codon 47 in *TP53* (Pro47Ser; P47S, rs1800371) was reported close to 30 years ago that alters proline at amino acid 47 to serine; data from our group showed that this SNP impairs phosphorylation on serine 46, which is critical for p53-mediated apoptosis (15). Subsequent data from our group and others have shown that the P47S variant is associated with increased cancer risk in mice (16) and humans (17), along with decreased response to most genotoxic chemotherapeutics (18). The underlying basis for the impaired tumor suppressor function of P47S has been linked to increased production of glutathione and antioxidants (19, 20), impaired ability to regulate ferroptosis (6, 16), increased activation of mTOR (21), impaired ability to regulate replication error restart (22), and impaired ability to recognize and bind to double-strand breaks in DNA (23). The P47S variant exists in approximately 6%–10% of Africans and 1%–2% of African Americans, so studies in this area have the potential to aid in the understanding of cancer risk disparities in this population. In addition, whereas our group discovered that P47S tumors are generally poorly responsive to genotoxic stress, we were able to show that these tumors possess enhanced sensitivity to agents like BRD2/4 inhibitors and cisplatin (24), thus establishing the paradigm that one can tailor chemotherapy to genetic variants of *TP53*.

There is emerging evidence that p53 plays a role in the regulation of the immune microenvironment (25, 26). For example, wild-type (WT) p53 can stimulate the M1, or proinflammatory, polarization of macrophages (27). Stabilization of WT p53 can also lead to increased tumor infiltration of T lymphocytes and tumor-suppressing immune activity (28). Conversely, mutant p53 can stimulate M2, or anti-inflammatory, polarization of macrophages, thereby contributing to immunosuppression (29, 30). For these reasons, and because of evidence for ethnic disparities in the response to immune checkpoint inhibitors (31, 32), we sought to determine the impact of the P47S variant on the tumor microenvironment (TME).

## Materials and Methods

### Cell Lines and Culture Conditions

Murine colon cancer cells derived from a female animal, MC38 (RRID: CVCL_B288), were obtained from the ATCC and grown in McCoy's 5A medium (Gibco) supplemented with 10% FBS (HyClone, GE Healthcare Life Sciences) and 1% penicillin/streptomycin (Corning Cellgro). Cells were grown in a 5% CO_2_ humidified incubator at 37°C. Cell line validation was completed using short tandem repeat profiling. Cells were tested for *Mycoplasma* contamination 1 week prior to use.

### Tumor Studies

All animal studies were performed in accordance with the recommendations in the Guide for the Care and Use of Laboratory Animals of the NIH. All protocols were approved by The Wistar Institute Institutional Animal Care and Use Committee. Hupki mice in a pure C57Bl/6 strain background [described previously (16)], homozygous for P47 (WT) or S47 *TP53,* between 6 and 10 weeks of age were used for all experiments. In most cases, the mice analyzed are littermate mice from P47/S47 heterozygous crosses; in all cases, P47 and S47 mice were no more than two generations removed from a backcross to each other. Mice were housed in plastic cages with *ad libitum* diet and maintained with a 12-hour dark/12-hour light cycle at 22°C. For tumor engraftment experiments, 1 × 10^6^ MC38 cells were injected subcutaneously into the right flanks of 6–10 weeks old male mice. For CD8^+^ T-cell depletion experiments, mice were treated with 200 μg anti-CD8b antibody (anti-mouse CD8b BioXCell, catalog no. BE0223) via intraperitoneal injection on the following schedule: 1 week prior to MC38 injections, 2 days prior to MC38 injections, two times per week for the duration of the study. For PD-L1 blockade therapy experiments, mice were administered three doses of anti-CD274 antibody (B7-H1, PD-L1; BioLegend: 124339) or three doses of rat IgG2b isotype control antibody (BioLegend: 400672) by intraperitoneal injection on the following schedule: 7 days post-MC38 injection, 10 days post-MC38 injection, 13 days post-MC38 injection. Tumor volumes were measured using digital calipers and tumor volume was calculated using the formula: volume = (length × width^2^) × 0.52. All mice were monitored daily for signs of pain or distress. Tumor volumes were measured every other day until the study endpoint, at which point all mice were euthanized.

### Bone Marrow–derived Macrophage Characterization

Bone marrow–derived macrophages (BMDM) were generated by culturing bone marrow cells from P47 and S47 mice with MCSF (BioLegend; 576406), as described previously (33). Bone marrow cells were cultured in DMEM with 10% FBS (Corning; 35-026-CV) and 100 U penicillin/streptomycin antibiotics in the presence of 40 ng/mL MCSF (BioLegend, 576406) at a density of 2–2.5 × 10^7^ cells/15 cm dish. After 5 days of differentiation, BMDMs were harvested using ice-cold detachment buffer containing: 1X PBS, 2% FBS, and 2 mmol/L etheylenediaminetetraacetic acid (EDTA) (Invitrogen; 15575-038) and used in polarization experiments. BMDMs were polarized to M1 phenotype by lipopolysaccharide (LPS) alone (100 ng/mL) or LPS + IFNγ (100 ng/mL; BioLegend, 575306) for 8 hours. BMDMs were polarized to M2 phenotype by IL4 (10 ng/mL; BioLegend, 574304) for 8 hours.

### Flow Cytometry

Spleens and tumors were harvested from P47 and S47 mice. Spleens were crushed through 70 μm cell strainers (Corning Falcon, 087712) using the plunger/piston end of the syringe, washed in 10 mL 1X PBS, resuspended in 10 mL of 1X red blood cell (RBC) Lysis Buffer (BioLegend, 420301), and incubated for 10 minutes to remove RBCs. RBC-depleted splenocytes were then washed with R10 medium [RPMI1640 (Millipore Sigma) containing 10% FBS (Gibco), 1% penicillin/streptomycin (Gibco, 15070-063), 1% GlutaMAX (Gibco, 35050-061), and 1% HEPES buffer (Corning, 25-060-CI)]. Excised tumors were washed in 10 mL 1X PBS and dissociated using the Tumor Dissociation Kit (Miltenyi Biotec; 130-096-730) according to the manufacturer's instructions. For staining surface markers, cell pellets were incubated with fluorochrome conjugated antibody cocktail for staining at 4°C for 30 minutes and were fixed using 1% paraformaldehyde (Sigma) for 20 minutes. All antibodies were used at 1:400 dilution. Samples were then washed and resuspended in 1x PBS and acquired on BD FACS Symphony flow cytometer. Data were analyzed using FlowJo v10 (RRID:SCR_008520, Treestar Inc.). Fluorochrome conjugated antibodies used for flow cytometry are listed in Supplementary Table S1.

### T-cell Stimulation Assays

Splenocytes derived from naïve, non–tumor-bearing S47 or P47 mice were adjusted to a concentration of 1 × 10^7^ cells/mL in R10 medium and 1 × 10^6^ total splenocytes were plated per well in a 96-well flat-bottom plate (Corning). Splenocytes were then either left unstimulated or stimulated with 1X (phorbol 12-myristate 13-acetate) PMA/Ionomycin (Cell Stimulation Cocktail, Invitrogen, 00-4970-93), 1X Concanavalin A (Invitrogen, 00-4978-93), or anti-CD3/CD28 Dynabeads (Gibco, 11141D) at a 5:1 bead to cell ratio. To measure degranulation, anti-CD107a antibody (clone 1D4B, BioLegend, 121610) was added to each well at the time of stimulation at a 1:200 concentration. After 1 hour of stimulation, 1X Brefeldin A (BioLegend, 420601) and 1X Monensin (BioLegend, 420701) were added to each well and cells were incubated for an additional 4 hours. After 5 hours of total stimulation, cells were washed, stained for surface antigens, and then stained for intracellular antigens using the BD Cytofix/Cytoperm Fixation/Permeabilization Kit according to the manufacturer's instructions (BD Biosciences, 554714).

### Dextramer Staining

Tumor-derived cells and splenocytes from tumor-bearing mice (TBM) were first washed and resuspended in FACS Buffer (1X PBS without Mg^2+^ or Ca^2+^, 2% FBS, 2 mmol/L EDTA) and stained in V-bottom 96-well plates. Cells were incubated simultaneously with TruStain FcX PLUS (anti-mouse CD16/32, BioLegend, 156604) and dextramers for the Rpl18 (KILTFD**R**L) neoantigen conjugated to APC (Immudex). Cells were incubated with Fc block and dextramers for 20 minutes at room temperature, then washed twice with FACS buffer before surface and intranuclear staining.

### Intranuclear Staining for Flow Analysis

Tumor-derived cells and splenocytes from TBM were first washed and resuspended in FACS Buffer (1X PBS without Mg^2+^ or Ca^2+^, 2% FBS, 2 mmol/L EDTA) and transferred to a V-bottom 96-well plate. After dextramer or other surface staining was performed, cells were washed in FACS buffer and fixed using the True-Nuclear Transcription Factor Buffer Set (BioLegend, 424401) according to the manufacturer's instructions specific to staining in 96-well plates.

### qRT-PCR

Mouse macrophage lysates were processed to obtain purified RNA using RNeasy Plus Micro Kit (Qiagen, 74034). RNA purified from lysates was used to synthesize cDNA using iScript Reverse Transcription Supermix kit (Bio-Rad, 1708841). RT-PCR reactions were carried out on Applied Biosystems Quant Studio 7 Flex Real-Time PCR system using 1X SYBR Green PCR Master Mix (Applied Biosystems). The relative mRNA expression was normalized to the expression of β-actin. Primer sequences are listed in Supplementary Table S2.

### ELISA

Supernatants from BMDM culture polarized with M1 stimuli were assessed for cytokine production by ELISAs. Commercially available kits from Invitrogen were used to measure IL6 (Invitrogen, 88-7064), IL12p40 (Invitrogen, 88-7120), and IL10 (Invitrogen, 88-7105) according to the manufacturer's instructions.

### Statistical Analysis

All statistical analyses between two groups were performed using the Wilcoxon rank-sum test when group sizes were small (<6 datapoints) or the Student *t* test when sample sizes were larger (e.g., Fig. 2), except for the analyses shown in Supplementary Fig. S1, where *P* values were determined by two-way ANOVA with *post hoc* multiple comparisons. Unless otherwise noted, all experiments were performed with three biological replicates (*n* = 3). All mouse experiments had a minimum of 5 animals/experimental group. Linear mixed models were used to analyze longitudinal tumor growth measures. Visualization and statistical comparisons of inhibitory receptor (IR) coexpression on tumor-derived T cells was performed using SPICE software (34). Wilcoxon rank-sum test was used to analyze area under tumor growth curve for PD-L1 immune blockade therapy experiments. All *in vivo* data are reported as mean ± SE. Statistical analyses were performed using GraphPad Prism 9.5.0 (RRID:SCR_002798).

### Data Availability

The data generated in this study are available upon request from the corresponding authors.

## Results

### S47 Exhibits Poorer Extrinsic Tumor Suppressor Function, Compared with Wt P53

**FIGURE 1:**
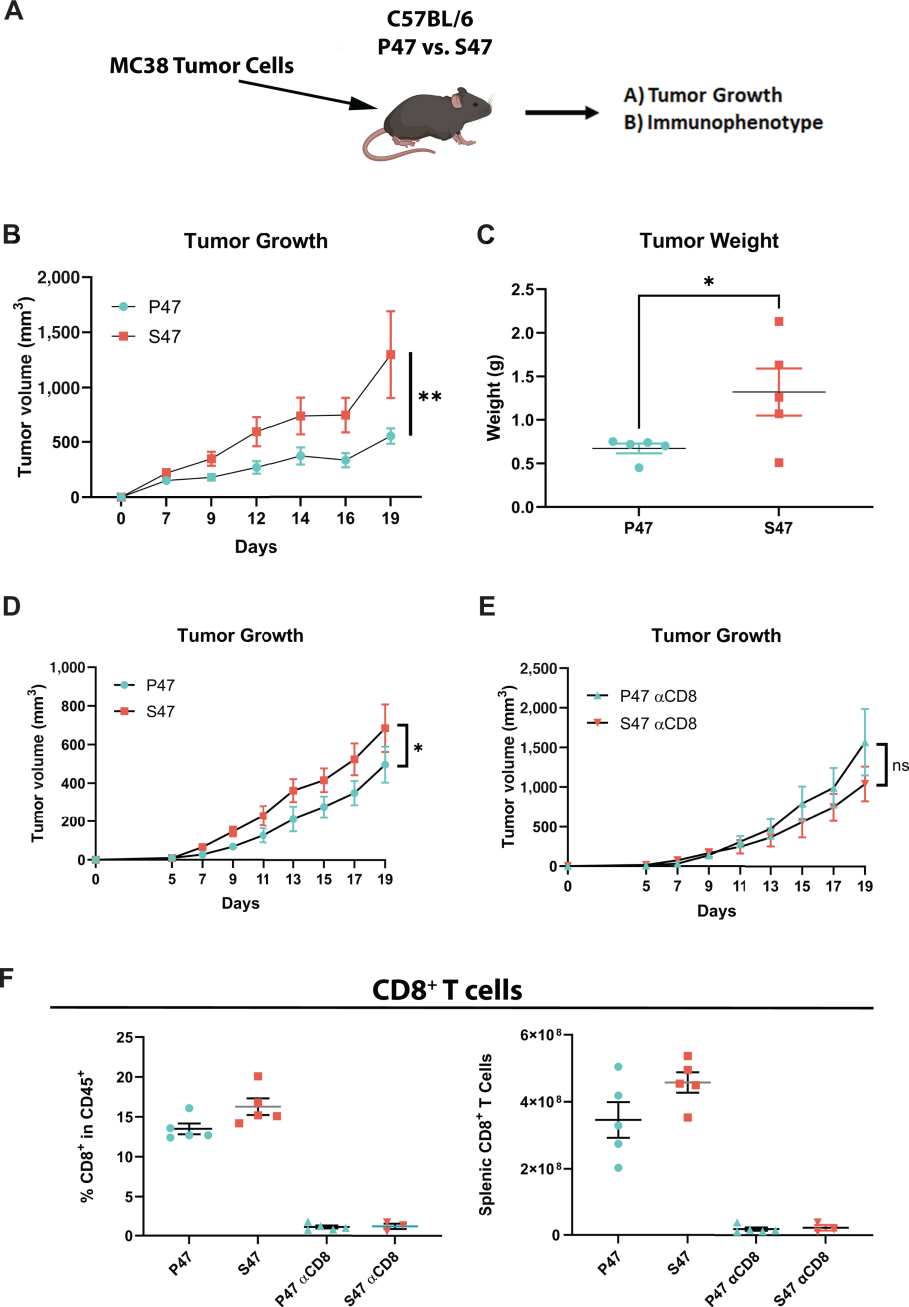
The tumor growth advantage in S47 mice is abolished following CD8^+^ T-cell depletion. **A,** Illustration of the experimental approach. Replicate P47 and S47 mice were injected subcutaneously with 1 × 10^6^ MC38 tumor cells. At day 19, spleens and tumors from mice were harvested and analyzed by flow cytometry. **B,** Tumor growth curves for P47 and S47 mice injected with MC38 cells. *n* = 5 mice per group. Linear mixed model used for statistical analysis. **, *P* < 0.01. **C,** Tumor weights of MC38 tumors harvested from P47 and S47 mice. *n* = 5 mice per group. Wilcoxon rank-sum test used for statistical analysis. *, *P* < 0.05. **D** and **E,** Tumor growth curves for P47 and S47 mice injected with MC38 cells, ± treatment with anti-CD8b antibody. *n* = 5 mice per group. Linear mixed model used for statistical analysis. *, *P* < 0.05. **F,** The percentage of CD8^+^ T cells of total T cells (CD45^+^/CD3^+^) in P47 and S47 mice before and after CD8^+^ T-cell depletion is shown on the left. Shown on the right are the total counts of splenic CD8^+^ T cells in P47 and S47 mice before and after CD8^+^ T-cell depletion.

We previously showed that the P47S variant of p53 (hereafter denoted S47) shows an intrinsic defect in tumor suppressor function and is associated with increased cancer risk (16, 24). In the current study, we sought to probe the impact of the P47S variant on the TME. To do this, the MC38 murine colorectal tumor cell line was injected subcutaneously into the flanks of mice of identical age and sex containing WT p53 (hereafter P47) or the S47 variant, and tumor growth was assessed over time (Fig. 1A). In two independent studies, we found a significant increase in MC38 tumor growth in S47 hosts, suggesting an extrinsic tumor suppressor defect for this variant (Fig. 1B and C). To distinguish whether this extrinsic defect was due to differences in adaptive immune function or other components of the TME, we performed a similar study and included experimental groups where both genotypes of mice were depleted for CD8^+^ T cells via administration of an anti-CD8b antibody. In the control groups, tumors again grew significantly larger in S47 hosts (Fig. 1D); however, this observed difference in tumor growth between genotypes was abolished when CD8^+^ T cells were depleted (Fig. 1E). Successful CD8^+^ T-cell depletion in the tissues was confirmed by flow cytometry (Fig. 1F). These data suggest that an altered immune microenvironment may underlie an extrinsic tumor suppressor defect in S47 mice.

### Basal Differences in the Level and Function of T Cells from S47 Mice

**FIGURE 2:**
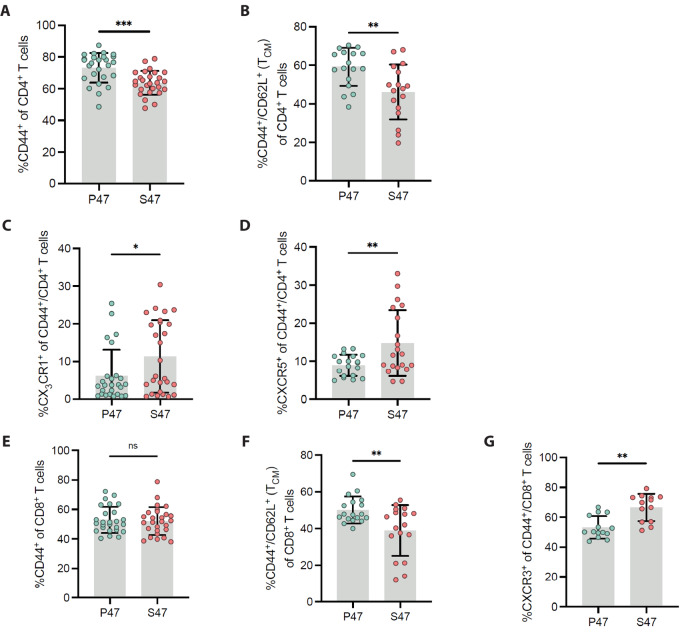
Differences in memory T-cell formation and chemokine receptor usage in S47 mice. Splenocytes derived from non–tumor-bearing, male S47 and P47 mice between the ages of 6 and 10 weeks were analyzed by flow cytometry for differences in baseline T-cell phenotypes. Data are derived from five independent cohorts of mice (*n* = 27 S47, *n* = 26 P47), and all panels incorporate multiple cohorts. **A** and **B,** Frequencies of total memory (CD44^+^) and central memory (CD44^+^/CD62L^+^) CD4^+^ T cells between P47 and S47 mice. **C** and **D,** Frequencies of CX_3_CR1, or CXCR5 chemokine receptor expression in memory (CD44^+^) CD4^+^ T cells between P47 and S47 mice. **E** and **F,** Frequencies of total memory (CD44^+^) and central memory (CD44^+^/CD62L^+^) CD8^+^ T cells between P47 and S47 mice. **G,** Frequency of CXCR3 chemokine receptor expression in memory (CD44^+^) CD8^+^ T cells between P47 and S47 mice. Statistics derived from two-tailed Student *t* tests. *, *P* < 0.05; **, *P* < 0.01; ***, *P* < 0.001.

We next sought to assess whether P47 and S47 mice possessed basal differences in immune cell phenotype and function. Toward this goal, we analyzed non-TBM of identical age and sex for the level of different subsets of immune cells. Because CD8^+^ T-cell depletion abolished the differences in tumor growth, and because we previously reported that S47 mice show increased populations of M2-like (anti-inflammatory) macrophages (35), we focused on these populations of immune cells. Our flow cytometric analyses revealed a significantly reduced population of memory (CD44^+^) CD4^+^ T cells in S47 mice (Fig. 2A). Specifically, we observed a significant reduction in central memory (CD44^+^ and CD62L^+^) CD4^+^ T cells in S47 mice (Fig. 2B). Conversely, we noted an increase in certain polarized mature CD4^+^ T helper subsets as evidenced by significantly elevated frequencies of CX_3_CR1^+^ and CXCR5^+^ CD4^+^ T cells in S47 mice (Fig. 2C and D), which are associated with Th1-polarized and T follicular helper cells, respectively. We found no evidence for differences in total memory CD8^+^ T cells between P47 and S47 mice (Fig. 2E); however, like the CD4^+^ T-cell compartment, we observed a significant reduction in CD8^+^ central memory T cells in S47 mice (Fig. 2F). Finally, we found evidence for a significant increase in activated CD8^+^ effector T cells in S47 mice, as evidenced by an increased frequency of %CXCR3^+^ CD8^+^ T cells (Fig. 2G). Our combined data are suggestive of a deficit in central memory T cell formation in S47 mice, accompanied by an increase in differentiated T helper and T effector subsets.

**FIGURE 3:**
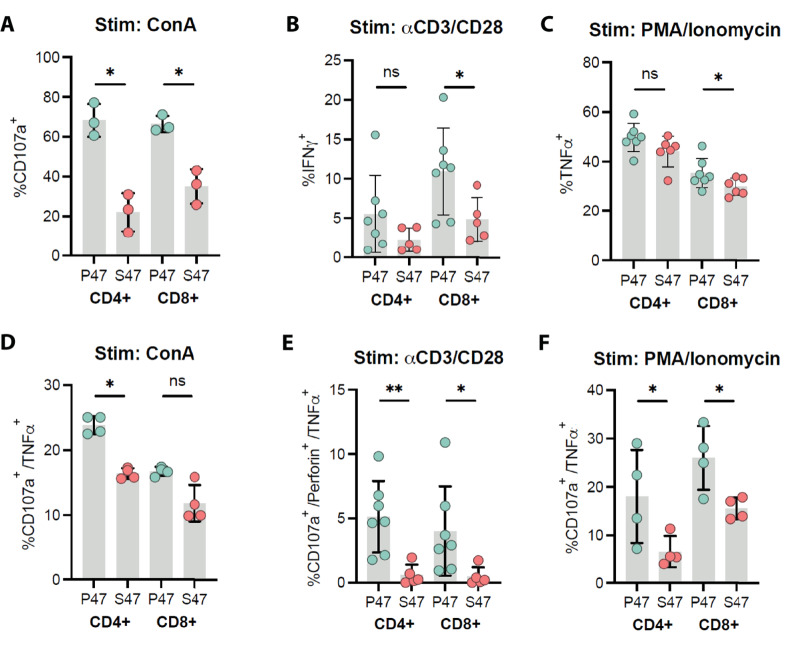
S47-derived T cells display a steady-state deficit in effector T-cell function after stimulation. RBC-depleted splenocytes from either S47 or P47 mice were stimulated with PMA/Ionomycin, Concanavalin A, or anti-CD3/CD28 Dynabeads, then assayed for cytolytic markers and effector cytokine production via multiparametric flow cytometry. Values were normalized to negative control wells receiving no stimulation. Data are derived from four independent experiments. **A–C,** Differences in CD4^+^ and CD8^+^ T-cell degranulation (CD107a), IFNγ production, and TNFα production between S47 and P47 mice when stimulated with Concanavalin A, anti-CD3/CD28 beads, or PMA/Ionomycin, respectively. Polyfunctionality profiles for CD4^+^ and CD8^+^ T cells double positive (CD107a^+^/TNFα^+^) when stimulated with ConA or PMA/Ionomycin (**D** and **F**, respectively) or triple positive (CD107a^+^/Perforin^+^/TNFα^+^) when stimulated with anti-CD3/CD28 beads (**E**) for effector molecules between S47 and P47 mice. Statistics derived from one-sided Wilcoxon rank-sum test. *, *P* < 0.05; **, *P* < 0.01; ***, *P* < 0.001.

We next assessed basal T-cell effector function in P47 and S47 mice. Upon stimulation with Concanavalin A (ConA), we found a significantly reduced frequency of degranulating (CD107a^+^) CD4^+^ and CD8^+^ T cells in S47 mice (Fig. 3A). These analyses also revealed a significant deficit in the production of effector cytokines, such as IFNγ and TNFα, from S47 CD8^+^ T cells, specifically in response to stimulation by anti-CD3/CD28 beads and PMA/Ionomycin (Fig. 3B and C). We next assessed polyfunctionality profiles of T cells derived from S47 and P47 mice after stimulation. We observed a significant reduction in CD107a^+^/TNFα^+^ T cells from S47 mice when stimulated with ConA or PMA/Ionomycin (Fig. 3D and F), and a reduction in CD107a^+^/Perforin^+^/TNFα^+^ T cells in S47 mice when stimulated with anti-CD3/CD28 beads (Fig. 3E). These combined data support the premise that T cells from S47 mice show reduced effector function following stimulation.

To extend these studies, we next analyzed macrophage function in P47 and S47 mice, by purifying BMDMs. We found evidence that upon stimulation, S47 BMDM exhibit lower levels of proinflammatory cytokines, IL6 and IL12p40, and higher levels of the anti-inflammatory cytokine IL10 (Supplementary Fig. S1A), along with an increase in *ARG1* expression (Supplementary Fig. S1B). These data are consistent with findings from our previous study, which indicated that S47 macrophages appear to be polarized toward an M2 immunosuppressive phenotype (35). Our combined data suggest the possibility of a more protumor immune microenvironment in S47 mice, evidenced from a basal decrease in S47 cytotoxic T-cell function following stimulation, along with a skewing toward an immunosuppressive macrophage phenotype.

### Increased Immunosuppressive Immune Cells in the Spleens of Tumor-bearing S47 Mice

**FIGURE 4:**
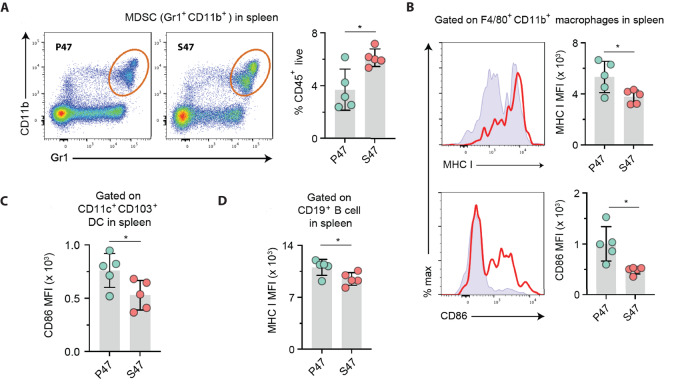
Tumor-bearing S47 mice harbor more immunosuppressive splenic immune cell profiles. **A,** Flow cytometry analyses on splenocytes from TBM for percent positive MDSCs in P47 and S47 mice, respectively. Bar graph on right shows the percent positive MDSCs out of the total CD45^+^ immune cells. *n* = 5 mice per group. **B,** Flow cytometry analyses of indicated activation markers on macrophages in the spleen. Histograms in the top show the mean fluorescent intensity (MFI) of MHC I and histograms in the bottom show MFI of CD86. The red solid line indicates P47 and the filled histogram indicates S47 sample. The bar graphs show quantitative data. *n* = 5 mice per group. **C** and **D,** Expression of CD86 and MHC I by CD11c^+^CD103^+^ DCs and CD19^+^ B cells, respectively, in spleens of TBM, shown as MFI. *n* = 5 mice per group. Statistics were derived from two-tailed Student *t* tests. *, *P* < 0.05.

It was next logical to identify any alterations in the immune systems of S47 TBM compared with TBM of equal age and sex with WT p53 (P47). To address this, we analyzed tumors and splenocytes from S47 and P47 TBM for immune cell distribution and activation phenotype by flow cytometry. We found no evidence for differences in the levels of populations of immune cells in the tumors of P47 and S47 mice, including myeloid-derived suppressor cells (MDSC), tumor-associated macrophages, dendritic cells (DC), or CD4^+^ and CD8^+^ T cells (Supplementary Fig. S2A–S2C). However, we noted significant differences in immune populations in the spleens of P47 and S47 mice. Specifically, we noted an increased population of MDSC in S47 TBM as determined by coexpression of the MDSC markers, Gr1 and CD11b, in CD45^+^ cells (Fig. 4A). In addition, the splenic macrophages from S47 TBM showed significantly lower levels of the activation markers MHC-I and CD86 (Fig. 4B). We also tested P47 and S47 splenocytes for the presence of activated DCs and B cells. These analyses demonstrated that S47 TBM possess significantly lower levels of activated DCs and B cells, compared with P47 mice (Fig. 4C and D). Taken together, these data are suggestive of a more immunosuppressive microenvironment in the spleens of S47 TBM, which could in turn serve to attenuate the tumor-specific adaptive cellular immune response.

### Evidence for Increased T-cell Exhaustion in S47 Tumor-infiltrating Lymphocytes

Our next goal was to determine whether there were any changes in the function of immune cells in tumor-bearing P47 and S47 mice. As stated previously, we found no significant differences in the frequencies of infiltrating immune cell subsets in tumors from P47 and S47 mice, suggesting no differences in trafficking of immune cells into tumors (Supplementary Fig. S2A–S2C). However, due to reduced levels of MHC class I and CD86 on antigen-presenting cells in S47 TBM, we interrogated whether the frequency of tumor-specific CD8^+^ T cells were significantly different between S47 and P47 TBM. Dextramer staining for CD8^+^ T cells specific for the KILTFD**R**L (KL8) neoantigen of MC38 tumor cells, located in the ribosomal protein L18 (36), demonstrated that there was no difference in the frequency of tumor-associated KL8-specific dextramer positive (KL8-dex^+^) CD8^+^ T cells in the TME between S47 and P47 mice, but that, unexpectedly, S47 mice harbored significantly elevated frequencies of KL8-dex^+^ CD8^+^ T cells in the spleen (Supplementary Fig. S2D). These latter data suggest that the CD8^+^ T-cell defect in S47 mice is unlikely to be due to differences in priming of tumor antigen-specific responses or to differences in trafficking of antigen-specific CD8^+^ T cells to the tumor.

**FIGURE 5:**
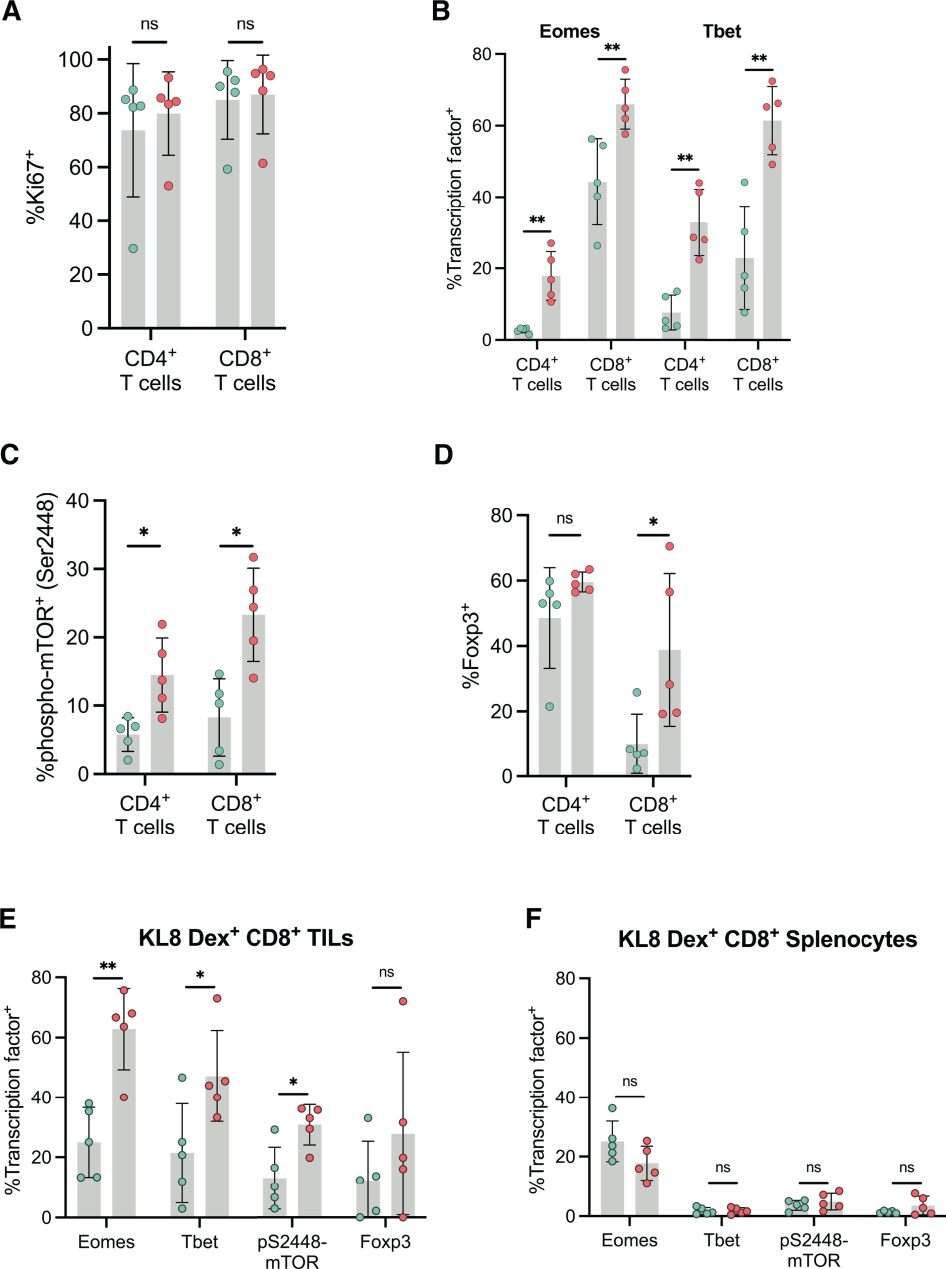
Tumor-infiltrating T cells in S47 mice display uniquely elevated transcription factor profiles. Tumors and spleens were harvested 21 days after injection of MC38 cells and T cells were analyzed for transcription factor expression by intranuclear flow cytometry. **A,** The percentage of proliferating (Ki67^+^) TILs in S47 and P47 mice. **B,** Frequency of Eomes^+^ or Tbet^+^ TILs in S47 and P47 mice. **C,** Frequency of TILs expressing mTOR phosphorylated at serine 2448 in S47 and P47 mice. **D,** Frequency of TILs expressing Foxp3 in S47 and P47 mice. Profiling of transcription factor expression by intranuclear flow cytometry of tumor-derived T cells from S47 and P47 mice. Frequency of tumor-derived **(E)** or spleen-derived **(F)**, KL8-dextramer^+^ CD8^+^ T cells expressing the Eomes, Tbet, phospho-S2448-mTOR, or Foxp3.

Next, we evaluated whether tumor-infiltrating lymphocytes (TIL) found in S47 TBM demonstrated alterations in transcription factor profiles or IRs, which would be indicative of attenuated functional capacity, when compared with P47 mice. There was no difference in the percentage of proliferating TILs as evidenced by Ki67 expression, indicating that S47-derived TILs are not anergic and respond to antigen (Fig. 5A). However, we did observe significantly altered transcription factor profiles in S47 TILs, with a higher frequency of CD4^+^ and CD8^+^ TILs expressing Eomes and Tbet in S47 TBM (Fig. 5B). Tbet phosphorylation and function have been linked to increased activation of the mTOR pathway (37), and we have recently published that S47 mice exhibit increased mTOR activity, compared with P47 mice (21). Consistent with this premise, in conjunction with elevated Tbet expression, we observed greater frequencies of phospho-Ser2448 mTOR^+^ TILs in S47 TBM compared with P47 TBM (Fig. 5C). We also found that S47 TBM exhibited significantly higher frequencies of CD8^+^ Foxp3^+^ TILs; a similar increase was not observed in the CD4^+^ T-cell compartment (Fig. 5D). Collectively, the elevated expression of Eomes, Tbet, and Foxp3 suggest an activated but potentially functionally-exhausted T-cell phenotype which may contribute to attenuated tumor clearance in S47 mice. Interestingly, the differences in transcription factor expression profiles between S47 and P47 TBM exhibited similar trends when analyzing KL8-dex^+^ CD8^+^ T cells derived from the tumor (Fig. 5E). In KL8-Dex^+^ CD8^+^ T cells derived from the spleen, transcription factor levels were globally reduced, with no significant difference between S47 and P47 mice (Fig. 5F), suggesting that differences in transcription factor profiles between S47 and P47 TILs are antigen-driven.

**FIGURE 6:**
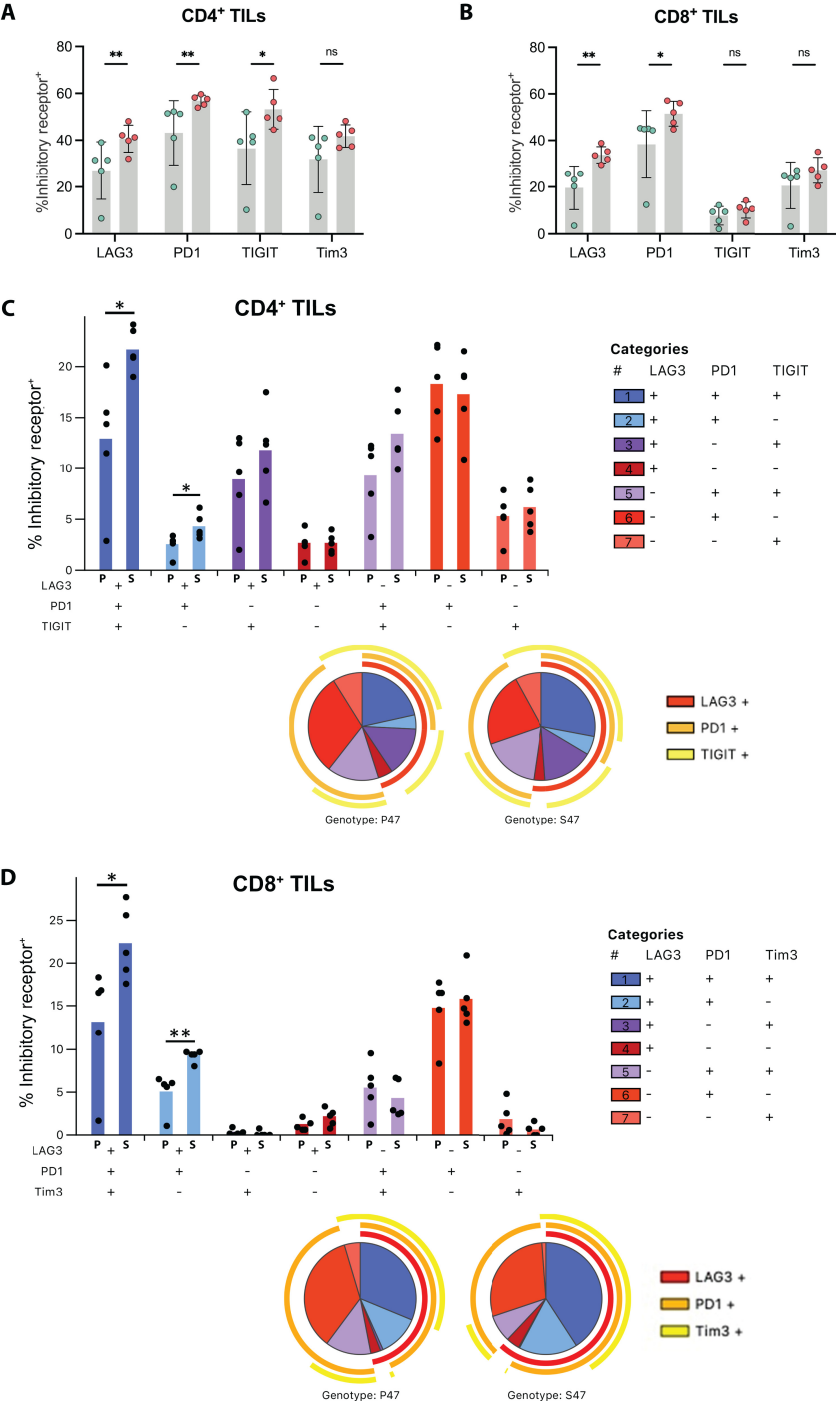
TILs in S47 mice exhibit elevated coexpression of multiple IRs. Tumors and spleens were harvested 21 days after injection of MC38 cells and T cells were analyzed for surface expression of multiple IRs by flow cytometry. Comparison of frequency of CD4^+^ TILs (**A**) and CD8^+^ TILs (**B**) expressing the LAG3, PD1, TIGIT, or Tim3 IRs between S47 and P47 mice. Comparison of the frequency of IR coexpression profiles in CD4^+^ TILs (**C**) and CD8^+^ TILs (**D**) between S47 and P47 mice. Bar graphs represent frequencies of specific IR coexpression profiles in the total CD4^+^ or CD8^+^ T cell populations derived from tumors, whereas pie charts represent relative frequencies of specific IR profiles in the total CD4^+^ or CD8^+^ TIL populations expressing any IR. Pie chart slices represent the average relative frequencies of 5 mice.

The expression of IRs in TILs is another major determinant of T-cell exhaustion (38). Therefore, we next analyzed the tumor-infiltrating populations of CD4^+^ and CD8^+^ T cells for expression of the IRs lymphocyte activating protein 3 (LAG3), programmed cell death protein 1 (PD1), T-cell immunoreceptor with Ig and ITIM domains (TIGIT), and T-cell immunoglobulin and mucin domain 3 (Tim3). We observed a significant increase in the frequency of LAG3^+^, PD1^+^, and TIGIT^+^ CD4^+^ TILs derived from S47 TBM, and a similar profile in CD8^+^ TILs with the exception of TIGIT which was globally lower on CD8^+^ TILs compared with CD4^+^ TILs (Fig. 6A and B). We next analyzed the coexpression pattern of IRs on CD4^+^ and CD8^+^ TILs between genotypes. TILs derived from S47 mice demonstrated an overall greater proportion of subpopulations that expressed more than one IR (blue/purple colors), and fewer subpopulations that expressed a single IR (red colors) when compared with P47 mice (Fig. 6C and D). This IR coexpression phenotype was dominated by a LAG3^+^/PD1^+^ population which was found in significantly higher frequencies in both CD4^+^ and CD8^+^ TILs from S47 mice (Fig. 6C and D, dark and light blue bars). Notably, the greatest proportion of IR^+^ TILs from S47 mice were triple positive for LAG3, PD1, and TIGIT or LAG3, PD1, and Tim3 in CD4^+^ and CD8^+^ TILs, respectively, and these populations were significantly enriched compared with P47 TBM (Fig. 6C and D, dark blue bars). These data support the premise that tumor-infiltrating T cells in S47 mice may be functionally impaired due to elevated levels of IR coexpression.

### Diminished Response of S47 TBM to Anti-PD-L1 Therapy

**FIGURE 7:**
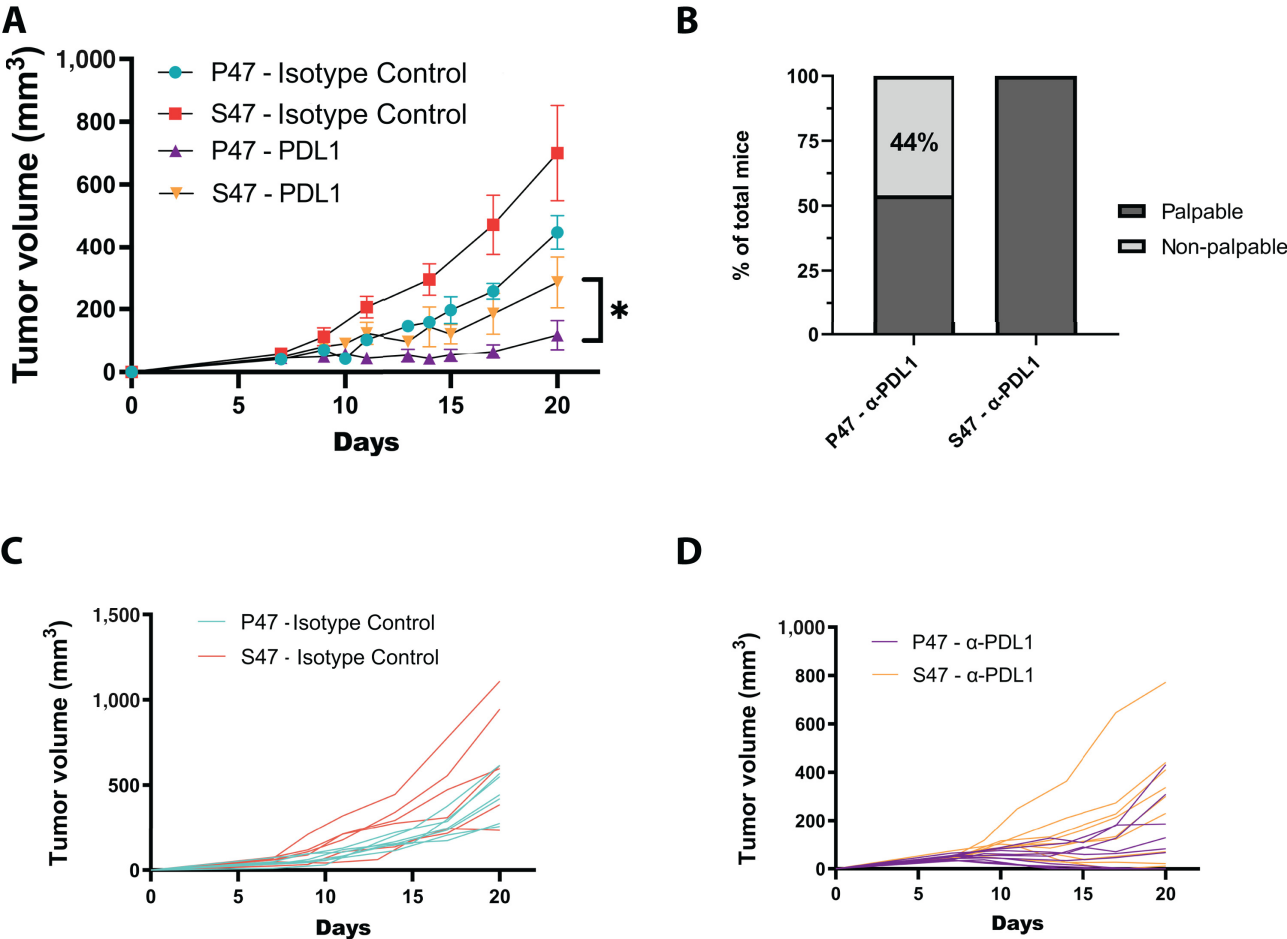
S47 mice are less responsive to PD-L1 blockade therapy. **A,** Tumor growth curves for P47 and S47 mice treated with rat IgG2b isotype control antibody or anti-PD-L1 antibody following MC38 cell injections. *n* = 7 P47 IgG2b isotype control; *n* = 5 S47 IgG2b isotype control; *n* = 9 P47 anti-PD-L1; *n* = 10 S47 anti-PD-L1. **B,** Graph showing the frequency of palpable tumors in S47 or P47 mice receiving anti-PD-L1 antibody. **C** and **D,** Individual tumor growth curves for each P47 and S47 mouse treated with rat IgG2b isotype control antibody or anti-PD-L1 antibody following MC38 cell injections. Statistics were derived from Wilcoxon rank-sum test. *, *P* < 0.05.

With the alterations in the immune microenvironment of S47 mice in mind, we next evaluated the efficacy of immune checkpoint blockade between genotypes. Toward this goal, we again analyzed MC38 tumor growth, as this tumor is known to respond to anti-PD-L1 therapy (39). We injected MC38 cells subcutaneously into P47 and S47 mice; after 7 days, when tumors were approximately 50 mm^3^, mice were randomly separated into two groups: one group received an IgG2b isotype control antibody and the other group received anti-PD-L1 antibody. Consistent with our previous results, tumors grew larger over time in S47 hosts compared with P47 (Fig. 7A). Both P47 and S47 TBM treated with anti-PD-L1 demonstrated tumor growth reduction when compared with genotype-matched isotype-treated controls, and when the increased growth rate of tumors in S47 hosts was taken into account, the overall fold decrease in tumor growth in P47 and S47 mice appeared similar (Fig. 7A). Notably, however, 4 of 9 P47 mice (44%) treated with anti-PD-L1 showed a complete response to anti-PD-L1 therapy, with no palpable tumors after three treatments (Fig. 7B–D). In contrast, all S47 mice had palpable tumors at the end of the study (0% complete response), indicative of a delayed or poorer response to this immune checkpoint blockade.

## Discussion

Previous work from our lab and others has shown that the S47 variant of *TP53* shows impaired “tumor-intrinsic” tumor suppressor function. This impaired tumor suppressor function has been attributed to reduced serine 46 phosphorylation (15), which is required for full p53-mediated apoptosis, as well as reduced sensitivity to ferroptosis (16), increased mTOR activity (21), decreased activity in replication error restart (22), and decreased ability to bind directly to sites of DNA damage (23). Herein, we show that this variant is associated with impaired “tumor-extrinsic” tumor suppressor function, as it is also associated with a more protumorigenic immune microenvironment. In our baseline immunophenotyping experiments, we found a deficit in the formation of central memory CD4^+^ and CD8^+^ T cells, and an increase in polarized CD4^+^ Th cells in S47 mice. Furthermore, upon stimulation, both CD4^+^ and CD8^+^ T cells from S47 mice show lower levels of degranulation, cytotoxicity, and effector cytokine production. Our analysis of BMDMs also demonstrates an anti-inflammatory/immunosuppressive phenotype in S47 mice. In TBM, our analysis of immune cell populations in the spleen shows an increase in MDSCs, along with decreased activation of macrophages, DCs, and B cells. Future studies are needed to more comprehensively analyze the differences in immune populations using single-cell RNA sequencing or other corroborative approaches.

With respect to tumor-specific T-cell responses, we did not observe reductions in the frequency of neoantigen-specific CD8^+^ T cells either in the spleens or tumors of S47 mice, suggesting that there are no overt deficits in priming or trafficking of S47 T cells specific for MC38-derived neoantigens. We note that the mismatch between the murine p53 expressed by the transferred MC38 tumor cells and the humanized p53 gene expressed by S47 and P47 mice in the Hupki background could lead to a significant murine p53-specific T-cell response, which was not measured in this study. However, given that a role for CD8^+^ T cells in suppressing MC38 tumor growth has been reported in C57Bl6 mice with fully murine p53 where a p53 mismatch is absent (40, 41), and that we have identified substantial frequencies of neoantigen-specific TILs in both P47 and S47 mice, it is unlikely that differences in a putative anti-murine p53 T-cell response between S47 and P47 mice are the primary driver of the differences in T-cell phenotypes between the p53 genotypes in this study. Nonetheless, this remains a possibility. Finally, we show evidence for increased markers of CD4^+^ and CD8^+^ T-cell exhaustion in S47 mice. All or many of these differences are likely to contribute to the increased tumor growth in S47 hosts, but the contribution of each of these parameters to the altered rate of tumor growth and differing efficacy of immunotherapy in S47 mice is likely to be complex. There have been other studies that have analyzed the impact of p53 loss or mutation on immune cell responses. However, most of the data published to date focuses on the immunologic outcomes associated with p53 knockout or “gain of function” mutants of p53 as opposed to hypomorphs that marginally affect p53 function. When p53 is absent in T cells only, there is a significant increase in T-cell effector functions (42), whereas p53 knockout in tumor cells is associated with a more suppressive tumor microenvironment (43, 44). This work is the first to show an impact of a naturally-occurring hypomorphic variant of p53 on the adaptive immune response.

Two issues are unresolved in this study. The first is whether the defects in immune cell function evident in our S47 mice also occur in humans. Interestingly, and consistent with our findings, increased markers of T-cell exhaustion have been reported in African-descent individuals (32). However, whether this defect is linked to the S47 variant remains to be determined. At present, genome-wide association studies (GWAS) have failed to identify the S47 variant as one that is associated with disease, most likely because of the low incidence of this variant and the paucity of sample numbers of African-descent individuals in GWAS databases. Whereas our previous case–control study found a significant association of P47S with premenopausal breast cancer, the effect was modest and therefore P47S is likely a low penetrance allele for breast cancer (17). Further studies evaluating P47S and the efficacy of immune checkpoint inhibitors in African-descent populations are needed, and we cannot rule out the possibility that the immune defects we see in mice may not be seen in humans.

A second issue unresolved in this study is the mechanism whereby the S47 SNP contributes to an altered immune microenvironment in mice. WT p53 has also been shown to activate immune-regulating genes such as TAP1 and CD80 (45, 46), and PD-L1 indirectly through its role in cellular senescence (47); while we have not identified these genes as differentially regulated in P47 and S47 cells, we cannot rule out the possibility that these genes could have impacted our results. Another possibility involves mTOR, the master regulator of metabolism. Our previous studies showed that, in mouse and human cells containing the S47 variant, there is decreased repression of the cystine transporter SLC7A11, leading to increased synthesis of the antioxidants glutathione and coenzyme A (19, 20). We showed that this increase in glutathione directly regulates the interaction of mTOR with Rheb; consequently, human and mouse cells containing the S47 variant show increased mTOR activity (21). For this reason, and given the prominent role of mTOR in immune cell differentiation and function (48), we monitored mTOR function in T cells from P47 and S47 mice and found evidence for increased mTOR activity (phospho-mTOR) in CD4^+^ and CD8^+^ TILs from S47 mice (Fig. 5C). mTOR has been found to stimulate the differentiation of effector T cells (49–51), and it inhibits the formation of CD8^+^ memory T cells (52, 53). Moreover, the increased expression of Tbet, a transcription factor controlling T-cell effector function, is also driven by mTOR activity (54). Overall, our data best support the conclusion that the efficacy of immunotherapy may be impacted by the S47 SNP, and that prolonged immunotherapy, and/or simultaneous targeting of multiple IRs, may be necessary for complete tumor regression in this population. Our study and our model serve as an important experimental platform upon which to test these hypotheses.

## Supplementary Material

Figure S1 Figure S1 shows BMDMs from S47 mice are more anti-inflammatory and polarize towards a M2 phenotype following IL-4 stimulation.

Figure S2 Figure S2 shows that the tumor microenvironment in both P47 and S47 mice is comprised of similar proportions of subpopulations of immune cells with similar phenotypes.

Table S1 and S2 Table S1 and Table S2
