# Supplementary material for: The African-centric P47S Variant of TP53 Confers Immune Dysregulation and Impaired Response to Immune Checkpoint Inhibition
Source: Cancer Res Commun. 2023 Jul 11;3(7):1200–11. doi: 10.1158/2767-9764.CRC-23-0149 (PMC10335007; doi:10.1158/2767-9764.CRC-23-0149)
Supplement: Figure S1 — shows BMDMs from S47 mice are more anti-inflammatory and polarize towards a M2 phenotype following IL-4 stimulation. [file crc-23-0149-s01.pdf]

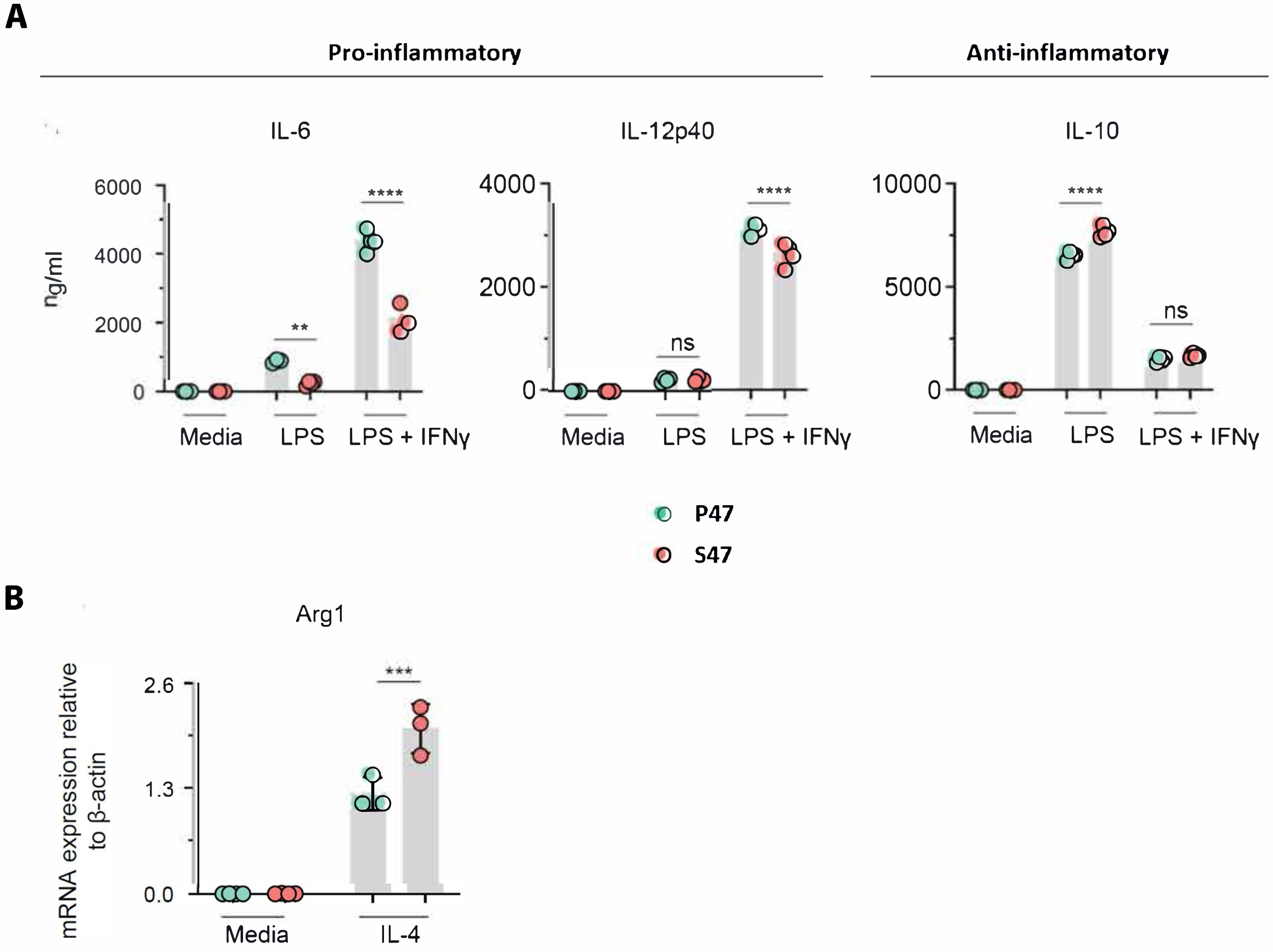

Figure S1: S47 macrophages polarize to an anti-inflammatory phenotype compared to P47 macrophages.
(A) ELISA showing measurements of IL-6, IL-12-p40, and IL-10 production by bone marrow-derived macrophages (BMDM) generated from P47 (n=4) or S47 (n=4) mice (green and red circles respectively), stimulated with LPS (100ng/ml) or LPS + INFγ (100ng/ml) for 8hr.
(B) RT-PCR analysis for the relative expression of Arg1 in BMDMs of P47 (n=3) and S47 (n=3) mice (green and red circles respectively) stimulated with IL-4 (10ng/ml) for 8hr. The mRNA relative expression is shown compared to β-actin.
Statistics were derived from two-way ANOVA with post-hoc multiple comparisons. *p<0.05, **p<0.01, ***p<0.005.
