# Supplementary material for: The African-centric P47S Variant of TP53 Confers Immune Dysregulation and Impaired Response to Immune Checkpoint Inhibition
Source: Cancer Res Commun. 2023 Jul 11;3(7):1200–11. doi: 10.1158/2767-9764.CRC-23-0149 (PMC10335007; doi:10.1158/2767-9764.CRC-23-0149)
Supplement: Figure S2 — shows that the tumor microenvironment in both P47 and S47 mice is comprised of similar proportions of subpopulations of immune cells with similar phenotypes. [file crc-23-0149-s02.pdf]

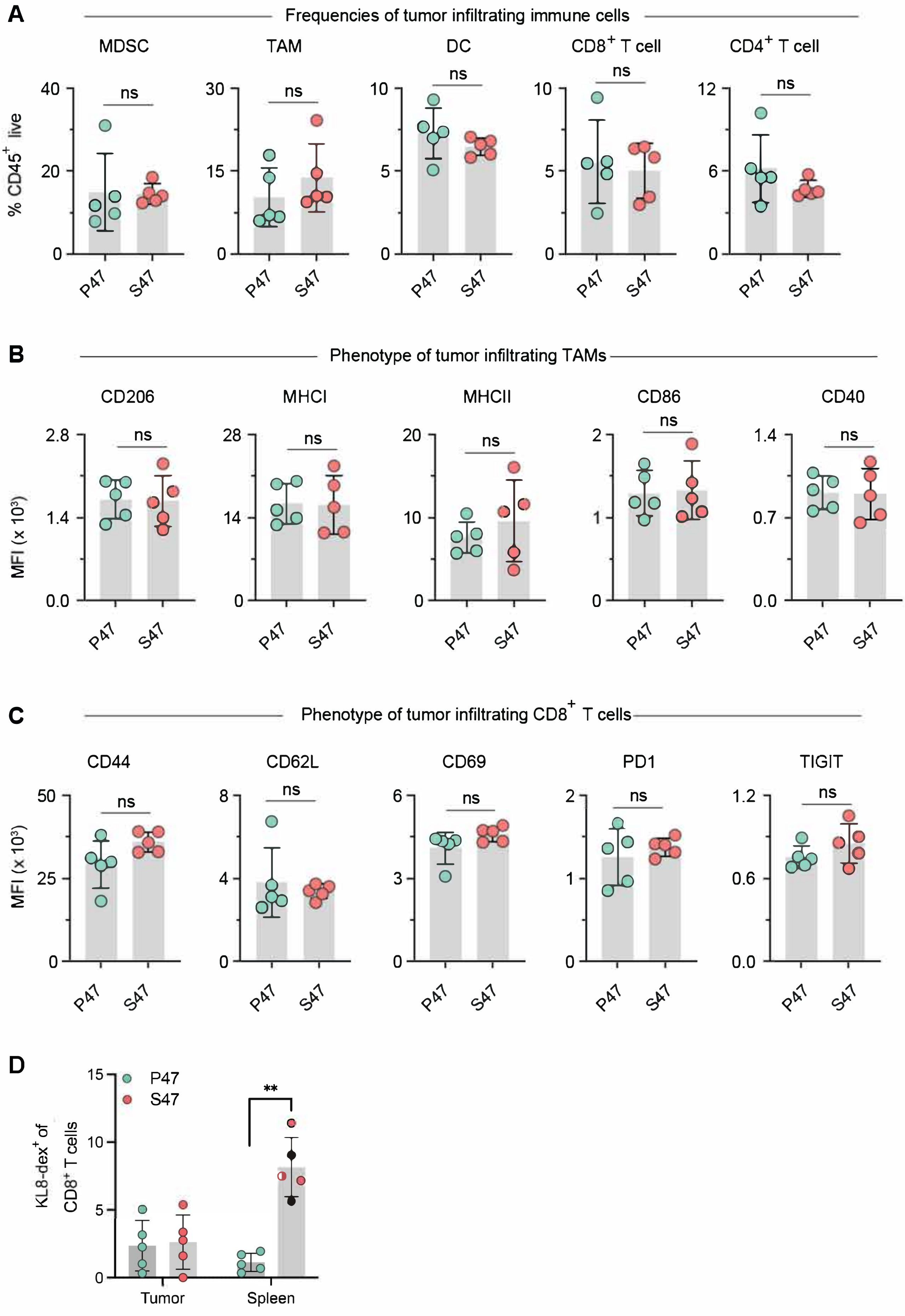

A
Frequencies of tumor infiltrating immune cells
MDSC
TAM
DC
CD8+ T cell
CD4+ T cell
% CD45+ live
ns
P47
S47
B
Phenotype of tumor infiltrating TAMs
CD206
MHCI
MHCII
CD86
CD40
MFI (x 10³)
C
Phenotype of tumor infiltrating CD8+ T cells
CD44
CD62L
CD69
PD1
TIGIT
D
KL8-dex+ of CD8+ T cells
**
Tumor
Spleen

**Figure S2: P47 and S47 mice do not show differences in frequencies and activation profile of immune cell infiltrates in the TME.**

**(A)** Flow cytometry analyses on tumor-infiltrating immune cells for MDSCs, TAMs, DCs, CD8+ T cells, and CD4+ T cells in P47 and S47 mice. n=5 mice per group.

**(B)** Flow cytometry analyses showing the activation profile of tumor-infiltrating TAMs as MFI for CD206, MHCI, MHCII, CD86, and CD40 in P47 and S47 mice. n=5 mice per group.

**(C)** Flow cytometry analyses showing the activation profile of tumor infiltrating CD8+ T cells as MFI for CD44, CD62L, CD69, PD1, and TIGIT in P47 and S47 mice. n=5 mice per group.

**(D)** Frequencies of KL8-dex+ CD8+ T cells in the tumors and spleens of P47 and S47 mice.

Statistics were derived from two-tailed Student's t-tests. *p<0.05, **p<0.01, ***p<0.005, ****p<0.001.
