# Supplementary material for: The African-centric P47S Variant of TP53 Confers Immune Dysregulation and Impaired Response to Immune Checkpoint Inhibition
Source: Cancer Res Commun. 2023 Jul 11;3(7):1200–11. doi: 10.1158/2767-9764.CRC-23-0149 (PMC10335007; doi:10.1158/2767-9764.CRC-23-0149)
Supplement: Table S1 and S2 — Table S1 and Table S2 [file crc-23-0149-s03.docx]

| **Supplemental Table 1: List of antibodies used for flow cytometry** | | | |
| --- | --- | --- | --- |
| Marker | Clone | Manufacturer | Catalog number |
| Zombie Aqua Viability Dye | 423102 | BioLegend | 423102 |
| TruStain FcX PLUS  (anti-mouse CD16/32) | S17011E | BioLegend | 156604 |
| CD3 | 17A2 | BioLegend | 100204 |
| CD19 | 6D5 | BioLegend | 115506 |
| CD45 | 30-F11 | BioLegend | 103147 |
| MHC-I | 34-1-2S | Invitrogen | 46-5998-82 |
| CD206 | MR6F3 | Invitrogen | 47-2061-82 |
| F4/80 | BM8 | BioLegend | 123110 |
| CD11b | M1/70 | BioLegend | 101257 |
| GR1 | RB6-8C5 | BioLegend | 108412 |
| MHC-II | M5/114.15.2 | BioLegend | 107632 |
| CD40 | 1C10 | Invitrogen | 15-0401-82 |
| PDL1 | 10F.9G2 | BioLegend | 124314 |
| CD86 | GL1 | BD Biosciences | 564199 |
| Ly6C | HK1.4 | BioLegend | 128041 |
| Ly6G | 1A8 | BD Biosciences | 741587 |
| CD11c | N418 | BioLegend | 117348 |
| CD103 | M290 | BD Biosciences | 741739 |
| CD8a | 53-6.7 | BioLegend | 100714 |
| TIM3 | RMT3-23 | BioLegend | 119704 |
| CD4 | GK1.5 | BioLegend | 100451 |
| CD44 | IM7 | BioLegend | 103012 |
| TIGIT | 1G9 | BioLegend | 142111 |
| CD107a | 1D4B | BioLegend | 121620 |
| PD1 | J43 | BD Biosciences | 744549 |
| CD69 | H12F3 | BioLegend | 104543 |
| CD62L | MEL-14 | BD Biosciences | 612833 |
| Ki67 | 16A8 | BioLegend | 652411 |
| CD44 | IM7 | BioLegend | 103010 |

| **Supplemental Table 2: List of primers used for RT-PCR** | | |
| --- | --- | --- |
| Gene name | Forward Sequence | Reverse Sequence |
| β-actin | CATTGCTGACAGGATGCAGAAGG | TGCTGGAAGGTGGACAGTGAGG |
| Arg1 | CATTGGCTTGCGAGACGTAGAC | GCTGAAGGTCTCTTCCATCACC |
